# Osteosarcoma of the Larynx: A Case Report and Review of Literature

**DOI:** 10.7759/cureus.39307

**Published:** 2023-05-21

**Authors:** Arup Ganguly, Rodolfo Garza, Vinayak Jain, Shravan Narmala

**Affiliations:** 1 Internal Medicine, Doctors Hospital at Renaissance/University of Texas Rio Grande Valley, Edinburg, USA; 2 Internal Medicine, MedStar Washington Hospital Center, Washington, DC, USA; 3 Hematology and Medical Oncology, Doctors Hospital at Renaissance, Edinburg, USA

**Keywords:** poorly differentiated carcinoma, rare cancers, total laryngectomy, carcinoma of larynx, osteosarcoma

## Abstract

The most common malignant laryngeal tumors are squamous cell carcinomas (SCCs), and other types such as sarcomas are rare. Osteosarcomas of the larynx are extremely rare within the subset of sarcomas, with very few cases reported in the literature. This cancer has a predilection for elderly males, in the sixth to eighth decades of life. Associated symptoms include hoarseness, stridor, and dyspnea. It is known to spread early and has a high rate of recurrence. We present the case of a 73-year-old male, a former smoker, who presented to the clinic with severe dyspnea and progressive hoarseness and was found to have a large exophytic mass arising from the epiglottis. A biopsy of the mass showed a poorly differentiated cancer with osteoid and new bone formation. He then underwent surgical removal of the mass, followed by radiation, and achieved clinical remission. However, a surveillance positron emission tomography (PET) scan 14 months later showed a hypermetabolic lesion in the left lung. Biopsy revealed metastatic osteosarcoma, and unfortunately, this cancer also spread to the brain. In this report, we will focus on the histological features of this rare malignancy and treatment options.

## Introduction

Malignant tumors of the larynx are relatively rare and are known to be either epithelial or non-epithelial in origin. Histologically, the most common tumor is squamous cell carcinoma (SCC) of the larynx, which accounts for roughly 90% of laryngeal malignancies [1]. Osteosarcomas of the larynx make up less than 1% of head and neck cancers. Most published literature indicates that this malignancy is associated with older males in the sixth to eighth decades of life and is not directly associated with tobacco smoking or alcohol exposure [2,3]. Since these tumors frequently arise from the vocal cords, they are commonly associated with hoarseness, dyspnea, and obstruction. They have the propensity to metastasize early via a hematogenous route, most commonly to the lung. To date, fewer than 40 cases of this malignancy have been reported in the literature [4].

## Case presentation

**Figure 1:**
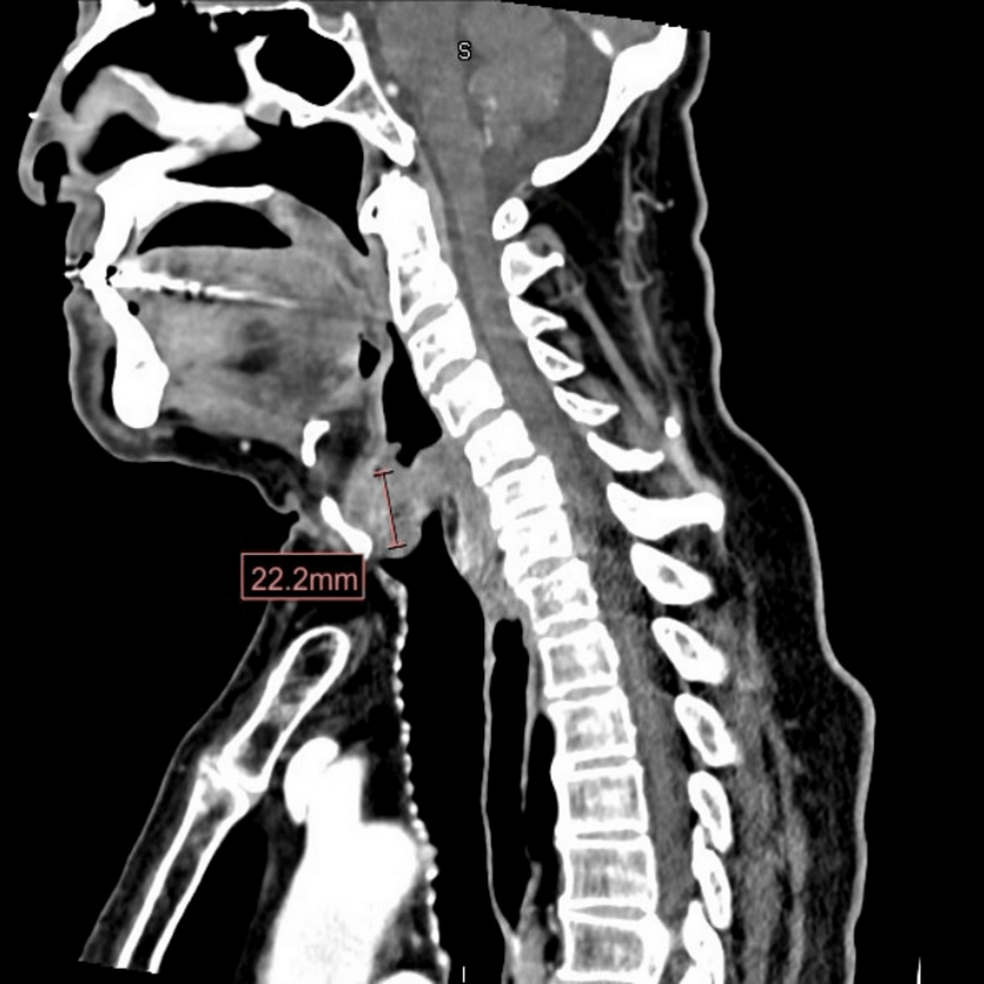
CT of the head and neck shows a lobulated mass centered along the anterior aspect of the larynx at the level of the vocal cords measuring approximately 2.2 × 1.4 cm CT: computed tomography

A 73-year-old male with a past medical history of hypertension presented with a four-month history of hoarseness and worsening dyspnea. The dyspnea was so severe that he reported difficulty sleeping at night. He endorsed a history of cigarette smoking having quit one month before presentation. Physical examination of the head, ear, neck, and throat was unremarkable. Computed tomography (CT) of the neck (Figure 1) done one week prior revealed a mass in the vocal cords.

**Figure 2:**
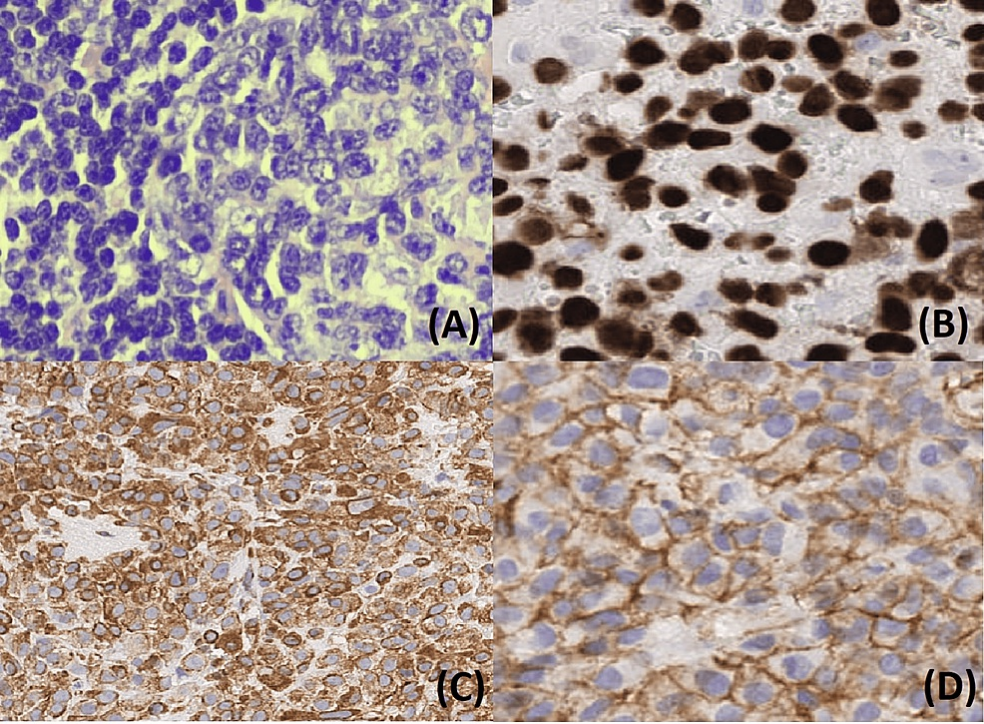
Immunohistochemistry analysis of the laryngeal mass Representative photomicrographs of the laryngeal mass showing (A) H&E stain and positive staining for (B) SATB2, (C) vimentin, and (D) CD99 H&E: hematoxylin and eosin

No suspicious lymphadenopathy was noted at that time. He then underwent microlaryngoscopy with intraoperative removal of the mass. On the fiberoptic examination, a large, irregular mass extending from the base of the epiglottis to the anterior commissure was seen. A specimen from the mass was sent for histopathologic analysis. The patient stated initially that his symptoms improved after the surgery. The final histopathology and immunohistochemistry report revealed a malignant neoplasm composed of sheets of cells with pleomorphic nuclei and prominent nucleoli with lace-like osteoid and new bone formation. Tumor cells were positive for SATB2, CD99, and vimentin (Figure 2) and were negative for AE1/AE3, p63, p40, desmin, S100, and SOX10. Given the characteristics of this immunohistochemical staining, the differential diagnoses included sarcomatoid carcinoma with heterologous osteosarcoma component or primary osteosarcoma, but a distinction could not be made.

**Figure 3:**
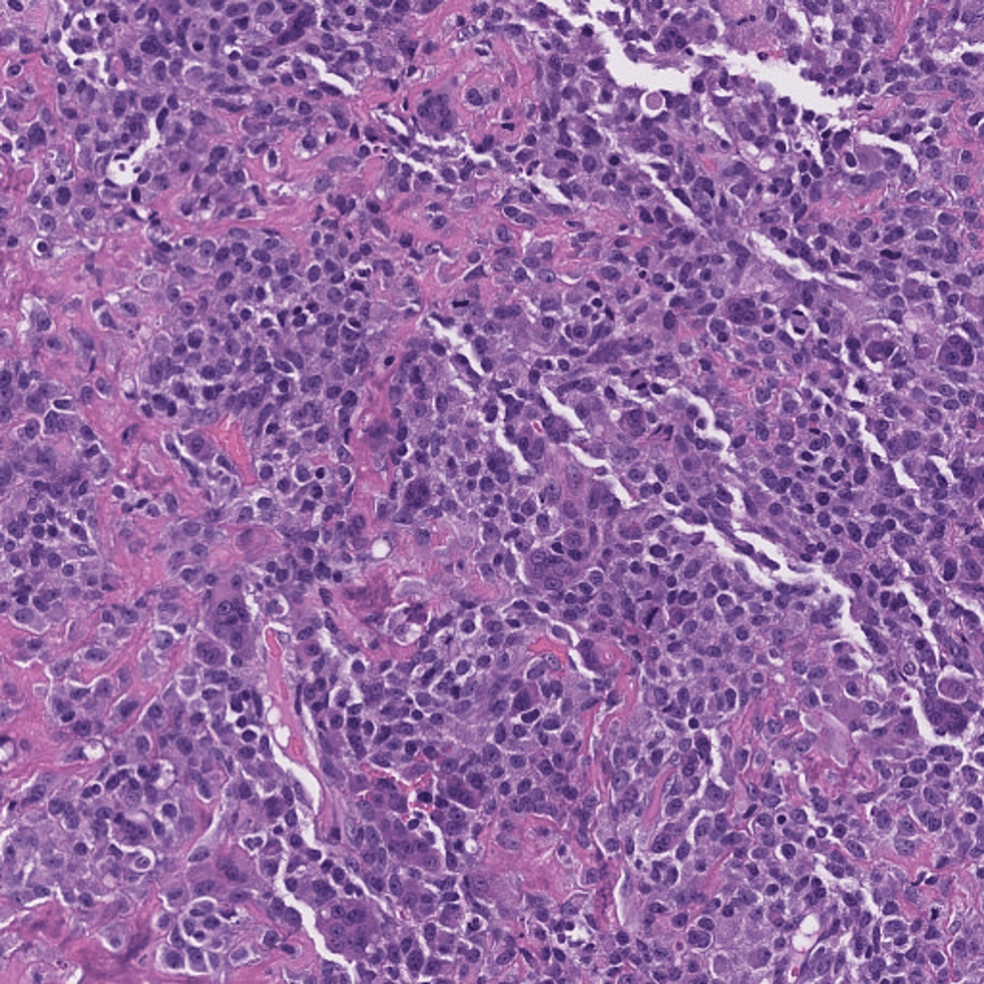
Repeat H&E stain of the mass showing a poorly differentiated tumor with highly atypical cells producing neoplastic osteoid H&E: hematoxylin and eosin

On a follow-up visit three days later, a repeat fiberoptic examination showed an irregular exophytic mass in the petiole of the epiglottis between the vocal cords in the same location as before but slightly lesser in size. There was rapid postsurgical growth with restriction of vocal cord mobility. The decision was then made for the patient to undergo a total laryngectomy with left neck dissection, right neck lymphadenectomy, total thyroidectomy with limited neck dissection, and cricopharyngeal myotomy, along with parathyroid exploration and reimplantation with a full body positron emission tomography (PET) scan afterward. However, before the agreed-upon date, he presented to the emergency room with acute dyspnea and stridor requiring emergent intubation for airway protection. The patient then underwent the planned surgery, and specimens from the larynx and surrounding lymph nodes were sent to the pathology laboratory. The histopathologic analysis confirmed a poorly differentiated tumor with highly atypical cells producing neoplastic osteoid (Figure 3).

When considering the previous pathology report, the diagnosis of osteosarcoma was preferred. The patient improved clinically and was eventually discharged with a tracheostomy tube in place. Whole-body PET scan showed increased uptake of radiotracer in the region to the left of the larynx of up to 9.2 SUV but no hypermetabolic focus suggestive of metastatic disease.

The decision was made to start adjuvant radiotherapy to improve chances of locoregional control, and he received a total dose of 6,000 centigray (cGy) delivered in 30 fractions over five weeks. No major side effects or interruptions occurred during this time. A repeat whole-body PET scan was performed three months after the last radiation dose and showed improvement from the previous scan and no evidence of metastatic disease. The patient also improved symptomatically, reporting increased appetite.

However, a surveillance PET scan in six months’ time showed the new appearance of a hypermetabolic calcified lesion in the left lung apex measuring 2.3 × 1.8 cm as well as the appearance of a right lower lobe hypermetabolic nodule. CT-guided biopsy of the right lung nodule was performed, which revealed metastatic osteosarcoma. During this time, the patient had no symptoms apart from fatigue. The options at this time were initiation of chemotherapy with cisplatin and doxorubicin versus gemcitabine/docetaxel versus cabazitaxel. However, before a decision regarding chemotherapy could be made, the patient developed right-sided weakness with altered mental status, and a CT of the head without contrast demonstrated a left frontal temporal intracranial hemorrhage. A magnetic resonance imaging (MRI) of the brain noted a large hematoma along with an 8 mm dural-based enhancing lesion in the right occipital lobe, consistent with metastasis. Given the extremely poor prognosis at that point, the patient’s family elected to pursue hospice care.

## Discussion

Malignant sarcomas comprise less than 1% of laryngeal neoplasms, and osteosarcomas are among the rarest of these tumors. Osteosarcoma is believed to develop from immature bone-forming cells or through the transformation of chondroblasts and fibroblasts into osteoblasts. Factors such as exposure to radiation, a previous history of retinoblastoma, Paget’s disease of bone, and fibrous dysplasia are considered possible causes. However, the specific etiologic factor for laryngeal osteosarcoma remains unclear, and there is no direct link between cigarette smoking or alcohol consumption and this type of cancer [2]. A physical examination will often reveal a mass in either the true vocal cords or the anterior commissure or the thyroid cartilage. The tumor commonly presents as either polypoid or exophytic in appearance, and its gross specimen can pose a challenge for cutting due to ossification. Additionally, the tumor tissue may exhibit a range of colors spanning from brown to red [4]. The tumor is made up of malignant spindle-shaped mesenchymal cells with neoplastic bone formation, exhibiting hyperchromasia, pleomorphism, and numerous mitosis. Microscopically, it may also have atypical giant cells, multinucleated osteoclast-like cells, and venous invasions. Immunohistochemical staining shows the neoplasm is positive for vimentin but negative for several markers, including desmin, S100 protein, and cytokeratin. Osteonectin and osteocalcin are expressed in osteogenic sarcoma and can distinguish osteoid from collagen matrix.

The most common approach to treatment draws principles from the treatment of both laryngeal carcinomas and osteosarcomas. In a recent study by Bennion et al. [5] that analyzed 33 reported cases of laryngeal osteosarcoma, surgery was the primary modality of treatment, with total laryngectomy being the most common approach [6]. Roughly 25% of patients had adjuvant radiation, and a similar percentage had chemotherapy as part of their primary treatment. The decision to provide radiation is usually individualized as these tumors tend to be radioresistant [7]. In our case, it was provided to achieve locoregional control as the mass was noted to be rapidly expanding, and the patient responded well to this treatment. Methotrexate, adriamycin, and cisplatin-based regimens are the most commonly used options for adjuvant chemotherapy, but their efficacy is controversial [7].

The use of more novel treatments such as immunotherapy may be of benefit in this type of cancer. The efficacy of immunotherapy in the treatment of osseous osteosarcoma has been studied. The use of immune checkpoint inhibitors targeting programmed cell death protein-1 (PD-1), cytotoxic T lymphocyte-associated protein 4 (CTLA-4), and programmed death ligand-1 (PD-L1) may offer benefits in the treatment of both primary and metastatic disease. The effects of PD-1 inhibitors, including pembrolizumab, nivolumab, and camrelizumab, the PD-L1 inhibitor atezolizumab, and the CTLA-4 inhibitor ipilimumab have been examined. However, in most of these trials, the drugs used showed little to no improvement in prognosis or the therapeutic effect was not observed [8]. There is, however, documented success in one instance of advanced metastatic osteosarcoma with nivolumab [9]. Chimeric antigen receptor T-cell (CAR-T) therapy has shown great promise in treating leukemias and lymphomas and may be a viable option for osteosarcomas. Several epitopes expressed by this tumor have been identified that may be potential targets for CAR-T, including human epidermal growth factor receptors (HER2) and disialoganglioside (GD2) receptors, among others [10,11]. There are no published studies or ongoing clinical trials that specifically investigate the efficacy of immunotherapies in the management of osteosarcoma of the larynx. In general, there is a need for further clinical trials that examine the effects of these novel treatments on both osseous and extraosseous osteosarcomas.

In the recent study by Bennion et al. [5], 36% of patients had not succumbed to their illness at the time of follow-up, which ranged between four and 96 months with disease-free survival in 28% of patients, the period for which also ranged from four to 96 months. Recurrence typically occurred within 12 months of diagnosis, and the most common site for distant metastasis was the lung. In the study by Mosalleum​​​​​​​ et al. [3], the two-year survival rate was found to be 23.5% with a mean survival rate of 12.6 months.

## Conclusions

Osteosarcoma of the larynx is an extremely rare entity with very few cases reported in the English literature. There are currently no standard guidelines on management, but the importance of surgical resection with adjuvant chemotherapy combined with early detection may prove to be quite beneficial. Although immunotherapy may be of benefit, further studies are needed to observe treatment outcomes and toxicities.
